# A Case of Large Vessel Giant Cell Arteritis Presenting With Cough and Diagnosed Using an FDG-PET Scan

**DOI:** 10.7759/cureus.59686

**Published:** 2024-05-05

**Authors:** Steven Danial Azmy Habib, Methsala Gunawardena

**Affiliations:** 1 Internal Medicine, Hampshire Hospitals NHS Foundation Trust, Basingstoke, GBR; 2 Rheumatology, Hampshire Hospitals NHS Foundation Trust, Basingstoke, GBR

**Keywords:** cough, large vessel vasculitis, giant cell arteritis (gca), 18f-fluorodeoxyglucose positron emission tomography (18f-fdg pet), methotrexate

## Abstract

Giant-cell arteritis (GCA) is a type of vasculitis characterised by the presence of granulomas. It is the predominant form of systemic vasculitis in adults and primarily affects the larger arteries in individuals aged ≥ 50 years. GCA affects the major arteries, such as the aorta and its branches, particularly the outer branches of the external carotid artery. Signs and symptoms can be categorised into cranial, extracranial, and systemic manifestations. Patients with headaches, jaw claudication, and vision disturbances usually have extracranial branches of the external carotid artery. Aside from being the prevailing manifestation of GCA, our primary concern regarding this variant is the potential for irreversible vision loss if not properly identified and addressed. Conversely, the GCA can also affect other major blood vessels such as the aorta.

Here, we present the case of a 70-year-old Caucasian female patient with cranial GCA who had experienced a temporal headache three years prior. The patient was successfully treated with prednisolone, which was gradually reduced to a very low level with the assistance of methotrexate. Recently, the patient presented with a dry cough that lasted for two months and elevated inflammatory markers. After thorough research, it was determined that there was no evidence of infection, including atypical infections, and that no abnormalities were found in the lungs. Ultimately, via an 18F-fluorodeoxyglucose-positron emission tomography (FDG-PET) scan, the patient was diagnosed with large vessel giant cell arteritis (LV-GCA). This impacted the aorta, carotid arteries, and subclavian arteries. The patient experienced notable improvement in her cough and a reduction in inflammatory markers after receiving a high dosage of oral prednisolone.

This case exemplifies the unusual manifestation of LV-GCA and verifies that recurring symptoms may differ from the original presentation. While dry cough is not commonly listed as a symptom of LV-GCA, it can be present as a manifestation or the sole presentation in certain patients, particularly when inflammatory markers are consistently high and there is no pulmonary disease.

## Introduction

Giant cell arteritis (GCA) is an immune-mediated inflammation that affects medium and large arteries. It is the most common type of systemic vasculitis in adults, with a higher occurrence in individuals aged ≥ 50 years. GCA pathogenesis is driven by T-cells and antigens, with different clinical subgroups likely arising from varying cytokine production [1]. Common symptoms of GCA include headaches, jaw pain, muscle aches, and visual problems. Ischemic consequences can lead to irreversible vision loss in 15-25% of patients if not identified and treated promptly, highlighting the urgent need for diagnosing GCA from an ophthalmological perspective [1,2].

Respiratory symptoms that affect both the upper and lower airways are sometimes observed. These symptoms include a dry cough, soreness of the throat and tongue, and hoarseness. These symptoms may go unnoticed in some patients, leading to a delayed diagnosis [3].

## Case presentation

A 70-year-old Caucasian female experienced polymyalgia rheumatica (PMR) in 2020, followed by giant cell arteritis (GCA) a few months later. The patient's symptoms improved with the administration of 60 mg of prednisolone once a day. Subsequently, the dosage of prednisolone was gradually reduced to 3 mg once a day and methotrexate to 15 mg once a week. The use of methotrexate temporarily ceased in June 2023 for two weeks before the patient underwent spinal fusion surgery for lumbar degenerative spondylosis. It was then resumed two weeks after the procedure. In addition, she had a medical history that included hypertension, glaucoma, and gastroesophageal reflux disease. A few weeks later, she expressed symptoms of dry cough, fatigue, difficulty breathing, excessive sweating throughout the night, loss of weight, and nausea persisting for two months. The patient was treated for a possible lower respiratory tract infection and was administered multiple rounds of antibiotics. In addition, the patient discontinued the use of methotrexate. However, there was no amelioration of the symptoms. She ruled out the potential for experiencing headache, visual impairment, pain in the jaw when chewing, sensitivity or pain in the scalp, discomfort in the joints, orthopnea, swelling in the legs, vomiting, or hemoptysis.

As a result, she was admitted to the hospital for a comprehensive medical assessment. Upon examination, her weight was 76 kg and her height was 170 cm. Her arterial pulses were palpable, with equal strength in all extremities. The patient's blood pressure was 158/79 and 155/77 mmHg in the right and left arm, respectively. The heart rate, respiration rate, and oxygen saturation levels were within normal ranges. She did not have a fever throughout the presentation or admission. The cardiovascular and respiratory examinations yielded normal results. Palpation of temporal artery pulses revealed normal findings. Abnormal test findings revealed a C-reactive protein level (CRP) of 129 mg/dl (normal range: 0-5) and an erythrocyte sedimentation rate of 123 mm/h (normal range: 0-20). Both have remained consistently elevated since she first had symptoms. The results of various laboratory tests, such as leukocyte count, liver function tests, kidney function tests, thyroid function tests, procalcitonin, anti-myeloperoxidase antibodies, anti-proteinase 3 antibodies, anti-GBM antibodies, connective tissue antibodies, allergens, immunoglobulins, TB screening, Aspergillus, and Aspergillus immunoglobulin G antibodies, were within the normal range or showed negative results. Computed tomographic (CT) scans of the thorax with pulmonary angiography revealed the absence of any pulmonary disease, pulmonary embolism, infarction, nodules, or aneurysm.

**Figure 1:**
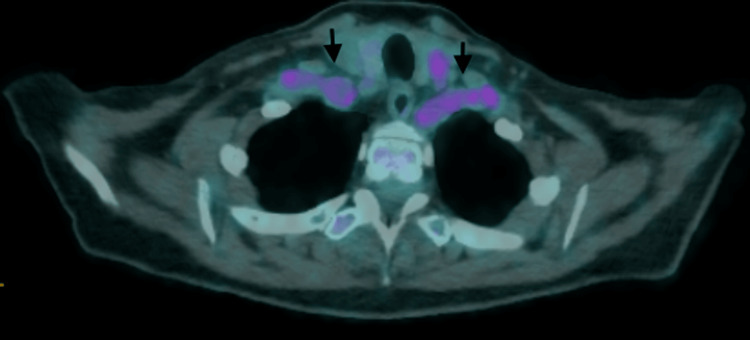
Bilateral increase in FDG absorption along the subclavian arteries (black arrows) FDG: 18F-fluorodeoxyglucose

**Figure 2:**
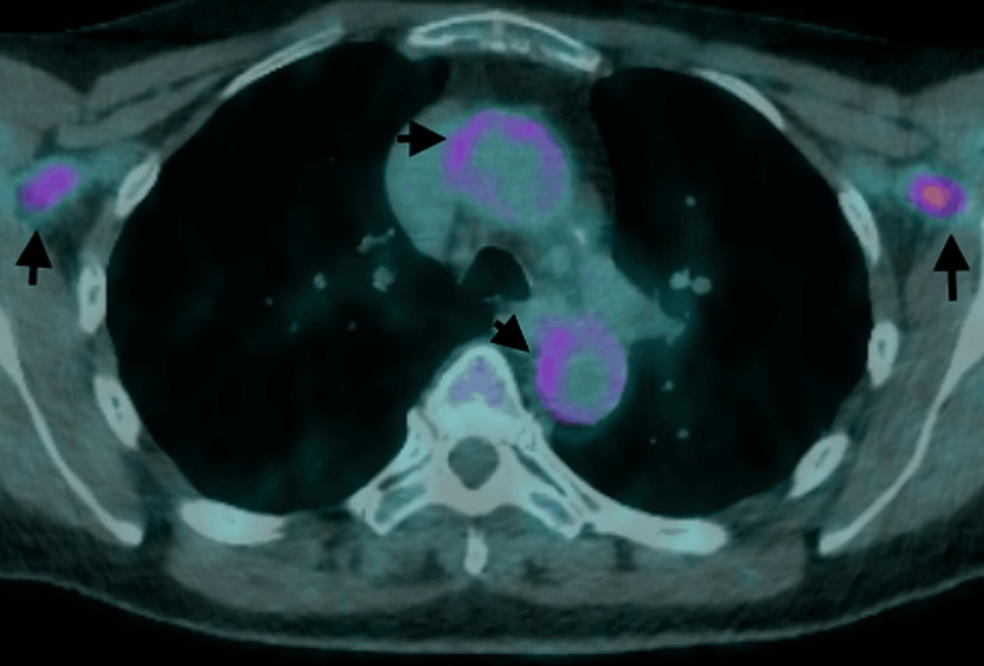
Enhanced FDG absorption along the ascending and descending aortas as well as axillary arteries bilaterally (black arrows) FDG: 18F-fluorodeoxyglucose

The presence of persistent symptoms, consistently high levels of C-reactive protein and erythrocyte sedimentation rate, the patient's medical history, and the absence of any abnormalities found in screenings and scans led to suspicion of large vessel giant cell arteritis (LV-GCA). Subsequently, an 18F-fluorodeoxyglucose positron emission tomography (FDG-PET) scan revealed heightened FDG activity in the aorta and subclavian arteries, as well as slightly increased FDG activity in both carotid arteries, indicating the presence of large-vessel vasculitis (Figure 1, Figure 2).

The symptoms and signs showed a quick response to prednisolone administration at a daily dose of 60 mg. The dosage was gradually reduced over subsequent weeks. The erythrocyte sedimentation rate decreased to 37 mg/dl, and the C-reactive protein level decreased to 2 mg/dl.

## Discussion

Cough is infrequently reported as an initial complaint in large-vessel giant cell arteritis (LV-GCA). Of the 88 patients diagnosed with giant cell arteritis (GCA), 12 experienced a dry cough. Among these 12 patients, 10 presented with other symptoms such as headaches and jaw claudication. In two cases, dry cough was described as the sole manifestation. The presence of dry cough in patients with large vessel giant cell arteritis (LV-GCA) was found to be associated with consistently higher levels of inflammatory markers as opposed to other clinical symptoms [4].

Pulmonary embolism, infarction, nodules, and pleural effusion are some instances of pulmonary complications resulting from GCA [5]. Cough often occurs in patients because of the involvement of the lung parenchyma or pleura. Nevertheless, our patient had a persistent cough without involvement of the lung tissue or lining of the lungs. Based on the close anatomical proximity of the vagal nerve to the aortitis, vagal hypersensitivity is hypothesised to be the cause. This hypothesis suggests that stimulation of the vagus nerve or one of its branches in the presence of inflammation in the aortic arch and aorta could lead to the observed symptoms. It is worth noting that there is no lung pathology associated with this condition [6].

A large number of patients are at risk of recurrence despite a positive response to corticosteroid therapy [7]. A cohort study was conducted on uniformly treated patients with GCA during a long-term follow-up. Despite the initially good response to corticosteroid treatment, 64% of the patients experienced a relapse in the current series. The most common symptom observed during relapse was polymyalgia rheumatica (PMR), which was reported in 51% of cases, followed by cranial symptoms, which were reported in 31% of cases [8]. In previous studies, headaches were the most prominent characteristic. This was followed by PMR and constitutional syndrome [9,10]. Accordingly, GCA recurrence can manifest in various separate forms, and in certain instances, the symptoms of recurrence may differ from the initial presentation. Constitutional syndrome is a frequently observed pattern of recurrence.

We speculate that our patient’s sudden relapse could have been caused by the omission of methotrexate. Although methotrexate cannot be used as an initial therapy instead of corticosteroids, it is effective in preventing relapses and increases the probability of achieving sustained discontinuation of corticosteroids. A meta-analysis was conducted using individual patient data from three randomised placebo-controlled studies involving individuals who were newly diagnosed with GCA. The treatment protocol involved administering high-dose corticosteroids initially, followed by the random administration of either oral methotrexate medication (7.5-15 mg/week) or a placebo. The adjunctive use of methotrexate in GCA decreases the likelihood of relapse and minimises exposure to corticosteroids. These data suggest that methotrexate could be a viable treatment option alongside corticosteroids in patients with GCA [11].

A cohort of patients with GCA was established at the outpatient clinic of the Hospital of San Carlos. The objective of this study was to evaluate the frequency and likelihood of relapses in patients diagnosed with GCA who were receiving treatment with or without methotrexate in clinical settings. This study demonstrated that the concurrent administration of methotrexate reduces the likelihood of relapses compared to those not receiving methotrexate. Patients receiving methotrexate benefit from the potential to rapidly reduce glucocorticoids, thereby minimising the potential for severe drug reactions associated with glucocorticoids. This study offers more evidence of the potential effectiveness of long-term methotrexate in treating individuals with GCA. It recommends initiating treatment with a minimum dose of 7.5-10 mg of methotrexate during the initial phases of the disease, along with prescribed doses of glucocorticoids [12].

**Figure 3:**
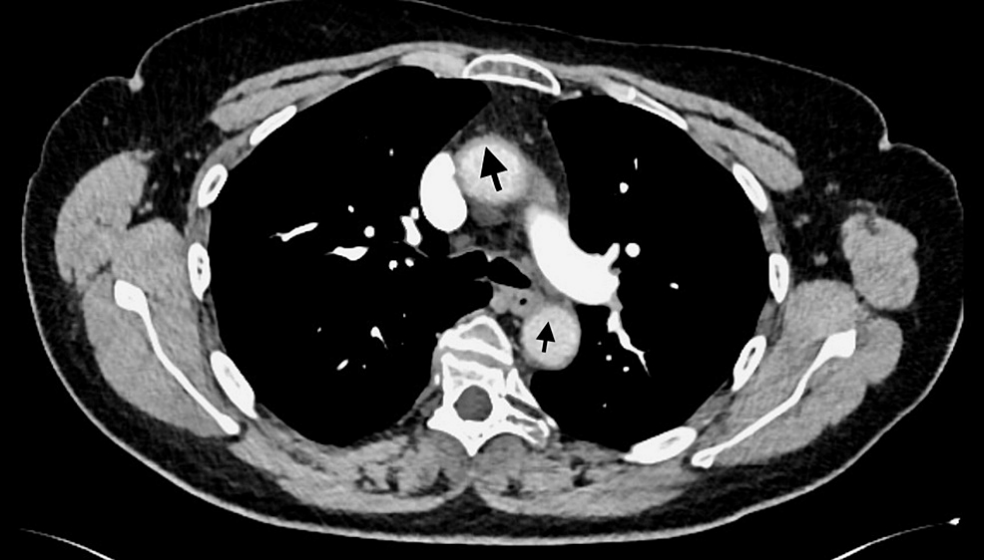
CT scan exhibiting subtle diffuse wall thickening of the ascending and descending aortas (black arrows)

**Figure 4:**
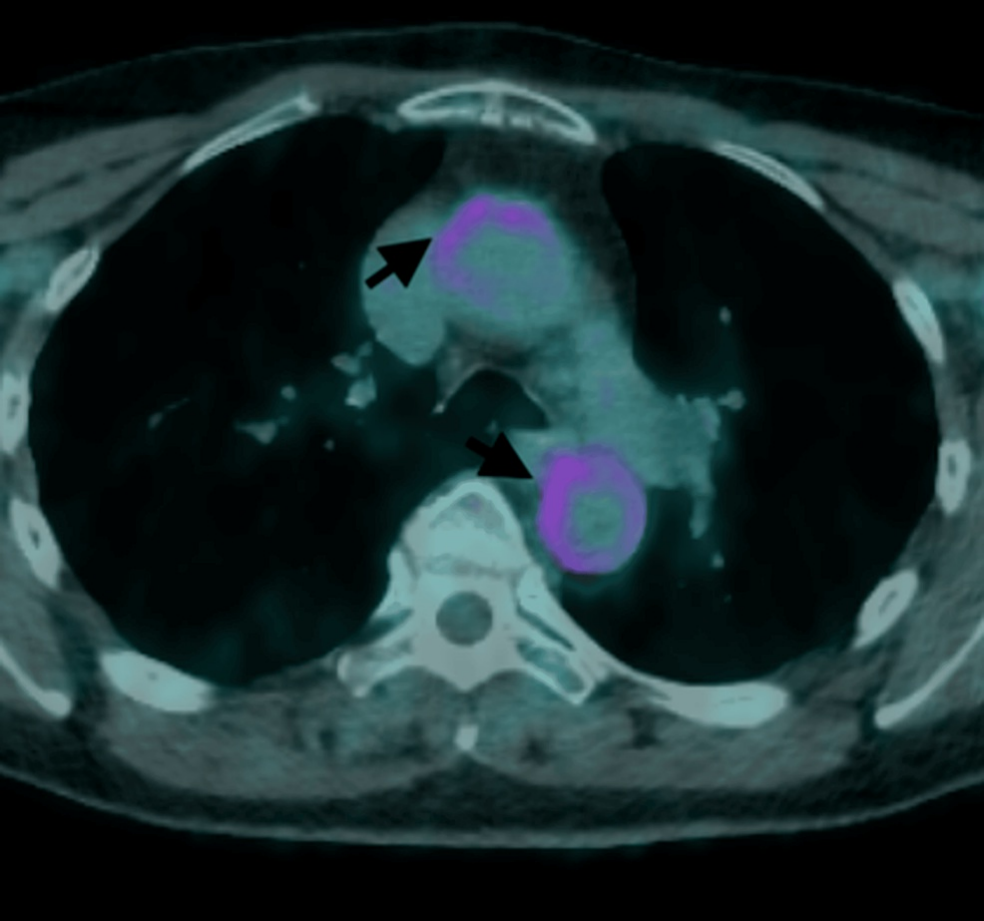
FDG-PET scan showing enhanced FDG activity in both the ascending and descending aortas (black arrows) FDG-PET: 18F-fluorodeoxyglucose-positron emission tomography

The specific imaging modality we are focusing on is 18F-fluorodeoxyglucose-positron emission tomography (FDG-PET), which is a functional imaging approach that is increasingly being used to diagnose inflammatory disorders such as sarcoidosis, vasculitis, and infections [13,14]. Studies have demonstrated that inflammatory cells, such as macrophages or granulation tissue, exhibit heightened glycolytic activity, leading to enhanced glucose uptake (or uptake of glucose analogues such as FDG), enabling the detection of aberrant metabolic activity in malignant and inflammatory cells (Figure 3 and Figure 4) [15]. Several studies have evaluated the utility of FDG-PET for the diagnosis of large-vessel vasculitis and its correlation with disease activity. These studies affirmed that FDG-PET is highly efficacious in evaluating both disease activity and the degree of large-vessel vasculitis [16,17]. Guidelines from the European League Against Rheumatism (EULAR) provide a thorough evaluation of different imaging methods for diagnosing and monitoring large-vessel vasculitis. According to these guidelines, FDG-PET has a significant benefit in its ability to identify alterations in the inner lining and wall thickness, along with the presence of other concurrent conditions, including infections and tumours. Therefore, it can be used in diagnosis and long-term monitoring [18].

## Conclusions

This case provides a clear example of the occasional deceptive presentation of GCA. Many patients display uncommon rather than more common symptoms. The patient's presentation during a recurrence may differ from that at the initial presentation. The significant presence of the patient's dry cough is noteworthy because respiratory symptoms can be a key indication of the disease, particularly in the presence of consistently high inflammatory markers and the lack of any pulmonary abnormalities, which can be verified by FDG-PET if clinically indicated. This facilitates early diagnosis, appropriate medication administration, alleviation of early symptoms, and prevention of adverse consequences.
